# Self-Reported Onset of Paroxysmal Atrial Fibrillation Is Related to Sleeping Body Position

**DOI:** 10.3389/fphys.2021.708650

**Published:** 2021-07-15

**Authors:** Lisa A. Gottlieb, Lorena Sanchez y Blanco, Mélèze Hocini, Lukas R. C. Dekker, Ruben Coronel

**Affiliations:** ^1^Institut de rythmologie et modélisation cardiaque (IHU Liryc), University of Bordeaux, Pessac, France; ^2^Department of Experimental Cardiology, Academic Medical Center, Amsterdam University Medical Center (AUMC), Amsterdam, Netherlands; ^3^Department of Cardiology, University Hospital, Bordeaux, Pessac, France; ^4^Department of Biomedical Engineering, University of Technology, Eindhoven, Netherlands; ^5^Department of Cardiology, Catharina Hospital, Eindhoven, Netherlands

**Keywords:** atrial fibrillation, questionnaire, body position, left lateral recumbence, body mass index

## Abstract

**Background:** Because stretch of the atrial myocardium is proarrhythmic for atrial fibrillation (AF) and a left lateral body position increases atrial dimensions in humans, we hypothesized that left lateral recumbence is a frequent AF-triggering body position in AF patients.

**Methods:** We performed a questionnaire study of symptomatic paroxysmal AF (episodes of AF < 1 week) patients scheduled for a first AF ablation therapy at Catharina Hospital, Eindhoven, the Netherlands and at University Hospital, Bordeaux, France.

**Results:** Ninety-four symptomatic paroxysmal AF patients were included [mean age 61 ± 11 years, median AF history of 29(48) months, 31% were females]. Twenty-two percent of patients reported a specific body position as a trigger of their AF symptoms. The triggering body position was left lateral position in 57% of cases, supine position in 33%, right lateral position in 10%, and prone position in 5% (*p* = 0.003 overall difference in prevalence). Patients with positional AF had a higher body mass index compared to patients without nocturnal/positional AF [28.7(4.2) and 25.4(5.2) kg/m^2^, respectively, *p* = 0.025], but otherwise resembled these patients.

**Conclusion:** Body position, and the left lateral position, in particular, is a common trigger of AF in symptomatic AF patients. Moreover, positional AF is associated with overweight. Understanding of the underlying mechanisms of positional AF can contribute to AF treatment and prevention.

## Introduction

Sleeping is a known trigger for atrial fibrillation (AF) and is considered to be caused by a high vagal nervous activity and obstructive sleep apnea (Rosso et al., 2010; Hohl et al., 2014). However the patient’s body position may also play a part in nocturnal arrhythmogenesis because a change in body position has been mentioned as a trigger for AF (Groh et al., 2019). It remains however unknown how often AF depends on body position and which body position is most prone to set off AF. Our objective was to evaluate the incidence of self-reported “positional” AF in symptomatic paroxysmal AF (episode duration <1 week) patients and the clinical characteristics of these patients to facilitate their identification in clinical practice. Because stretch of the atrial myocardium is proarrhythmic for AF (Ravelli and Allessie, 1997), and a left lateral body position increases atrial dimensions in humans (Wieslander et al., 2019), we hypothesized that left lateral recumbence is a frequent AF-triggering body position. AF treatment and prevention can be ameliorated by understanding the underlying mechanisms of positional AF.

## Materials and Methods

**Figure 1:**
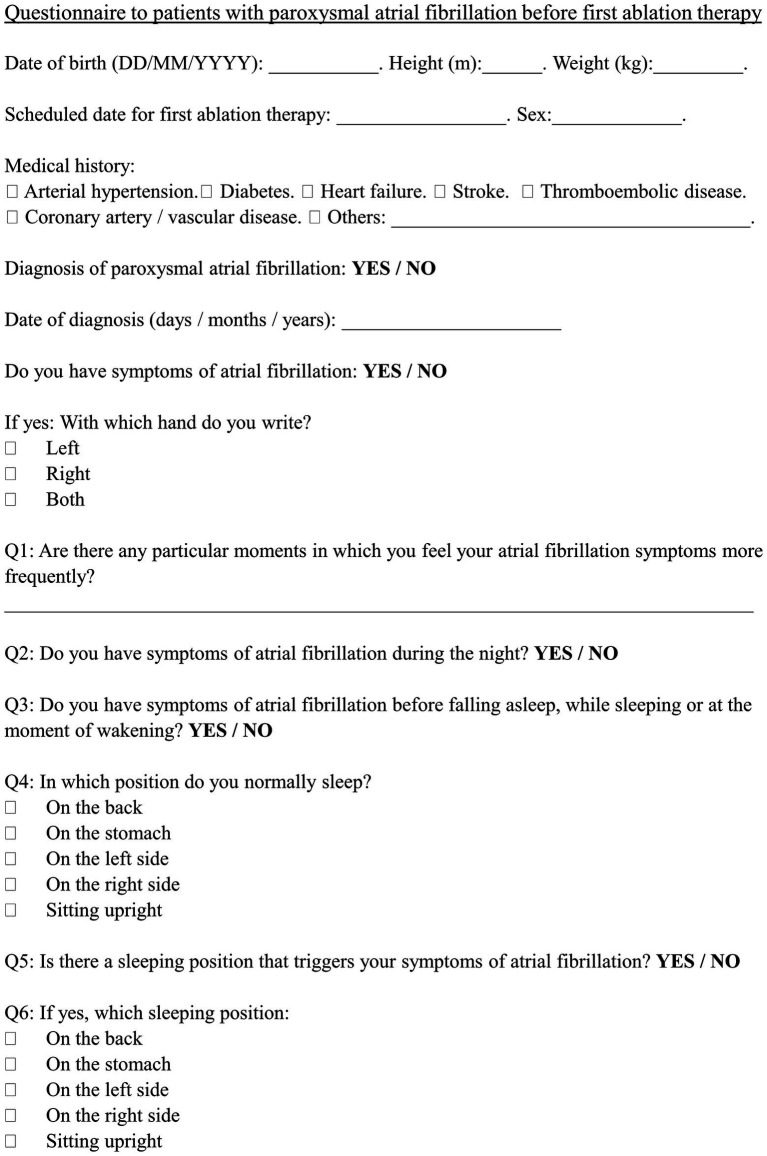
Questionnaire. A 1-page questionnaire was handed out to drug-resistant paroxysmal AF patients admitted to a tertiary center before elective ablation therapy.

A questionnaire (Figure 1), including medical history, clinical characteristics and symptom-triggering situations, was handed out to drug-resistant symptomatic paroxysmal AF patients electively hospitalized before a first ablation therapy. The patients anonymously and voluntarily responded to the questionnaire based on their recollection within 24 h of the scheduled ablation procedure. Patients from two tertiary centers (Catharina Hospital, Eindhoven, the Netherlands and University Hospital, Bordeaux, France) were asked to participate over the time course of 3 months at each center. The Medical Research Involving Human Subjects Act does not apply to this study that was considered by the relevant ethical boards as part of the standard taking of a medical history.

Nominal data are expressed as mean ± standard deviation and median (interquartile range) dependent of normality evaluated by a Shapiro–Wilk’s test. Differences in clinical characteristics between patient groups with positional AF, nocturnal AF (but not positional AF), and non-positional/non-nocturnal AF were tested with an ANOVA or a Kruskal-Wallis analysis, as appropriate. A *post hoc* correction (Bonferroni or Tukey) for multiple testing was applied. A *χ*^2^ goodness of fit test was used to estimate the difference in prevalence of self-reported AF-triggering body positions.

## Results

Ninety-four patients filled out the questionnaire (*n* = 36: Catharina Hospital, Eindhoven, the Netherlands; *n* = 58: University Hospital, Bordeaux, France). The average age was 61 ± 11 years, 31% were female, and AF diagnosis was made 29(48) months earlier. Hypertension had been diagnosed in 32% of patients.

**Figure 2:**
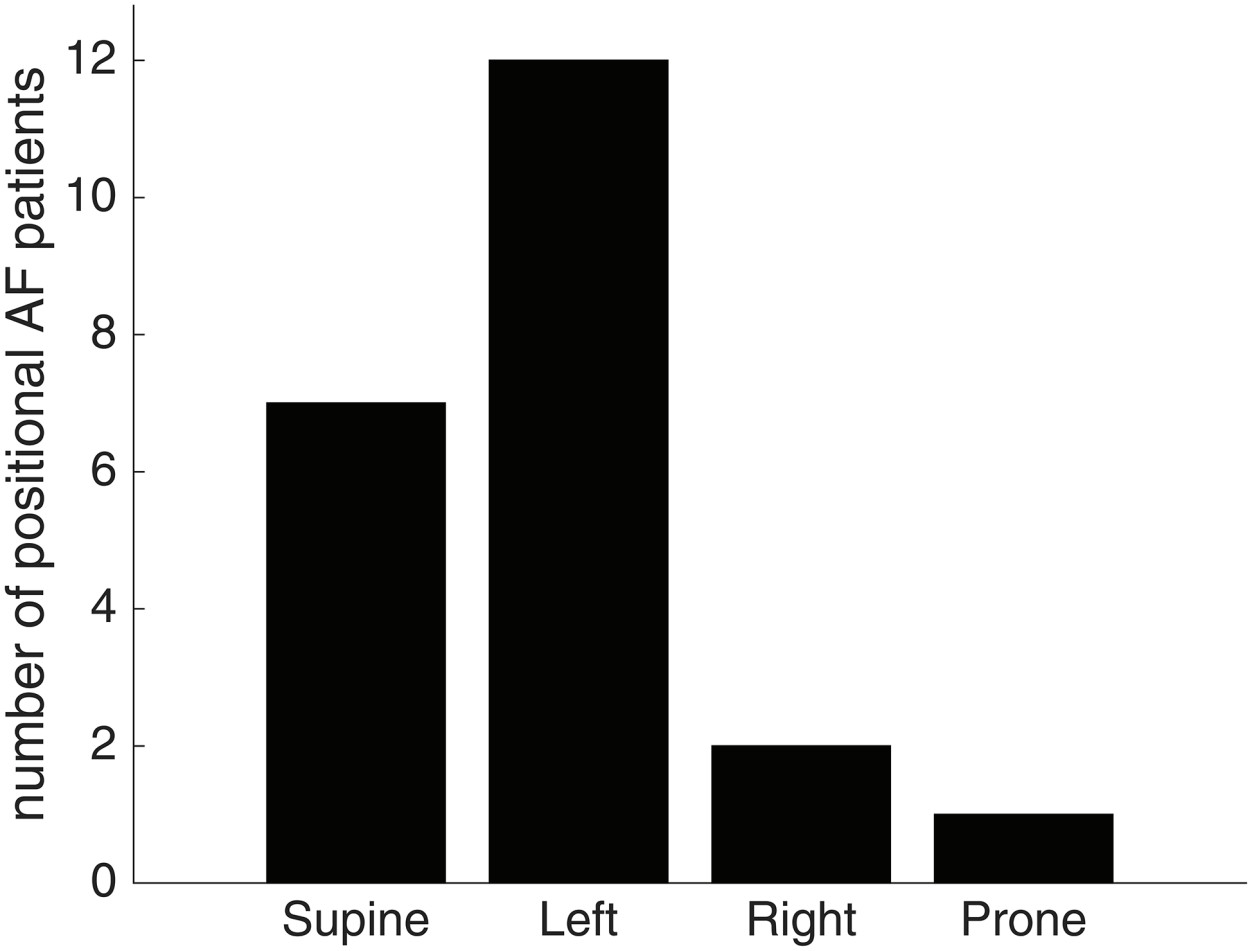
The self-reported AF-triggering body position in patients with positional AF. Twenty-one patients reported that taking a specific body position triggered their AF symptoms. The most AF-triggering body position was the left lateral and supine position. A *χ*^2^ goodness of fit test demonstrated a statistically significant difference in the prevalence of AF-triggering body positions (*p* = 0.003). One patient described both the left and right lateral body position as symptom triggering.

AF symptoms during the night, at the moment of falling asleep, or upon awakening occurred in 77% (72/94) of patients (Table 1). Twenty-two percent (21/94) of patients reported that a specific body position triggered their AF symptoms. The AF-triggering body position was the left lateral position in 57% (12/21), the supine position in 33% (7/21), the prone position in 5% (1/21), and the right lateral position in 10% (2/21; *χ*^2^ goodness of fit test: *p* = 0.003; Figure 2). One patient described both the left and right lateral body position as symptom triggering.

**Table 1:** Self-reported triggering situations of atrial fibrillation (AF) symptoms.

Self-reported AF trigger	*N* (%)
Sleep-related	72 (76.6)
Body position	21 (22.3)
Exercise	21 (22.3)
Stress/anxiety	13 (13.8)
Postprandial period	5 (5.3)
Alcohol intake	3 (3.2)
Fatigue	3 (3.2)
Following exercise	3 (3.2)

Sleep-related situations (moment before falling asleep, upon awakening, during rest, and night time), body position, and exercise were the most common self-reported trigger situations of AF symptoms in 94 drug-resistant paroxysmal AF patients. Several patients reported multiple AF triggers.

Patients with positional AF had a higher body mass index (BMI) compared to patients without nocturnal/positional AF [28.7(4.2) and 25.4(5.2) kg/m^2^, respectively, *p* = 0.025], but otherwise resembled these patients (Table 2). The BMI was similar in patients with supine and left lateral positional AF [28.7 (3.7) and 28.6 (5.5) kg/m^2^, respectively; *p* = 0.298].

**Table 2:** Medical history, clinical characteristics, and preferred sleeping positions.

		Positional AF	Nocturnal AF	Non-nocturnal /non-positional AF
Patients, *n*		21	51	22
Age, years		62 ± 11	60 ± 12	64 ± 10
Female sex, *n* (%)		7 (33.3)	17 (33.3)	5 (22.7)
BMI, kg/m^2^		28.7 [4.2]*	27.5 [5.4]	25.4 [5.2]
AF duration, months		48 [67]	24 [52]	33 [36]
History of	Arterial hypertension, *n* (%)	7 (33.3)	15 (29.4)	8 (36.4)
	Coronary artery disease, *n* (%)	2 (9.5)	2 (3.9)	2 (9.1)
	Transient ischemic attack/cerebral vascular apoplexy, *n* (%)	3 (14.3)	3 (5.9)	1 (4.6)
	Diabetes mellitus, *n* (%)	1 (4.8)	3 (5.9)	0 (0)
	Heart failure, *n* (%)	1 (4.8)	1 (2.0)	1 (4.6)
	Thromboembolic disease, *n* (%)	2 (9.5)	2 (3.9)	1 (4.6)
Preferred sleeping position	Supine position, *n* (%)	6 (28.6)	15 (29.4)	8 (36.4)
	Prone position, *n* (%)	2 (9.5)	6 (11.8)	3 (13.6)
	Left lateral position, *n* (%)	11 (52.4)	30 (58.8)	12 (54.6)
	Right lateral position, *n* (%)	10 (47.6)	27 (52.9)	12 (54.6)
	Sitting, *n* (%)	0 (0)	1 (2.0)	0 (0)

Patients with positional AF (*n* = 21) were more overweight compared to patients without self-reported nocturnal/positional AF (*n* = 22; asterisk: *p* = 0.025) but resembled otherwise these patients. Arterial hypertension was a frequently reported comorbidity in all patient groups. The preferred sleeping positions did not differ between the three groups of paroxysmal AF patients. Lateral recumbence during sleeping was preferred in all groups. Several patients stated multiple preferred sleeping positions. Data are expressed as mean ± standard deviation or median (interquartile range) dependent on normality tested with a Shapiro–Wilk’s test. An ANOVA or Kruskal-Wallis analysis was applied to test for differences between the three patient groups. Statistical significance marked with asterisk.

Patients with nocturnal AF (but not positional AF) preferred similar sleeping positions compared to patients with positional AF and to patients with non-nocturnal/non-positional AF (Table 2).

## Discussion

We report that positional AF is common in drug-resistant symptomatic paroxysmal AF patients and is associated with overweight. A left lateral or supine position triggered AF symptoms in most of these patients. Moreover, nocturnal AF occurred in the majority of patients and these patients preferred similar sleeping positions as patients without nocturnal symptoms.

A left lateral recumbent position increases the dimensions of the left atrium and the right pulmonary veins and thereby increases local myocardial stress (Wieslander et al., 2019). Stretch of the atria and pulmonary veins is proarrhythmic for AF (Ravelli and Allessie, 1997; Chang et al., 2007). Because the pulmonary veins are fixed to the mediastinum, we speculate that a body position change contributes to AF genesis by augmenting wall stress in the pulmonary veins.

Increased parasympathetic nervous activity is known to be proarrhythmic for AF (Chen et al., 2014) and may mediate an arrhythmogenic effect in left lateral recumbence. However, although a decrease in heart rate occurs in the left lateral position in an elderly population, heart rate variability as a measure of autonomic nervous modulation was similar to in supine position (Sasaki et al., 2017). Alternatively, the thorax shape may change in lateral recumbence compared to supine position thereby causing intrathoracic pressure changes that stimulate cardiopulmonary baroreflexes.

Obstructive sleep apnea is an underdiagnosed risk factor for AF that causes intrathoracic pressure changes that cyclically augments atrial wall stress (Young et al., 1997; Joosten et al., 2014). Apneic episodes often occur in supine position (Joosten et al., 2014). The patients with supine positional AF may suffer from undiagnosed (sub)clinical apneic episodes during sleep. The association between positional AF and overweight may be explained by proarrhythmic mechanical alterations in the thorax, similar to in the setting of apnea. Obesity and obstructive sleep apnea are indeed associated comorbidities (Gami et al., 2007). Moreover, overweight itself is associated with atrial mechanical changes because a higher prevalence of a left atrial hypertension exists in overweight and obese AF patients compared to patients with normal BMI (Sramko et al., 2017). Additionally, adipose tissue in the atria promotes AF by causing structural remodeling and electrical conduction abnormalities (Hatem et al., 2016).

## Limitations

Our objective was to evaluate self-reported positional triggers of AF symptoms, and the study was therefore by design descriptive. Verification of symptoms is not possible. However, future polysomnographic studies can evaluate the effect of body position on triggering of asymptomatic AF and quantify the temporal relation between body position change and AF onset. A right–left side confusion may have influenced the self-reported AF-triggering body position. The questionnaire included an early question about handedness in order to prime the patient’s right–left awareness. Finally, self-reported retrospective data are subject to recall bias. The prevalence of positional AF may therefore be underestimated.

## Conclusion

Body position, and the left lateral position, in particular, is a common trigger of AF in symptomatic AF patients and is associated with overweight. We propose that patient specific AF-triggering body positions are included in the diagnostic work-up and that the effect of lateral body position on atrial electrophysiology and wall stress is assessed in paroxysmal AF patients. Thus, AF treatment and prevention can be ameliorated by understanding the underlying mechanisms of positional AF.

## Data Availability Statement

The raw data supporting the conclusions of this article will be made available by the authors, without undue reservation.

## Ethics Statement

The Medical Research Involving Human Subjects Act does not apply to this study that was considered by the relevant ethical boards as part of the standard taking of a medical history.

## Author Contributions

LG: conceptualization, methodology, software, validation, formal analysis, investigation, data curation, and writing - original draft. LB: conceptualization, methodology, investigation, data curation, and writing - review and editing. MH: conceptualization, validation, supervision, and writing - review and editing. LD and RC: conceptualization, funding acquisition, validation, supervision, and writing - original draft. All authors contributed to the article and approved the submitted version.

### Conflict of Interest

The authors declare that the research was conducted in the absence of any commercial or financial relationships that could be construed as a potential conflict of interest.
